# Law and Medicine

**Published:** 1900-03

**Authors:** C. A. F. Lindorme

**Affiliations:** Chicago, Ill.


### LAW AND MEDICINE.

By C. A. F. LINDORME, Ph.D., M.D.,

Chicago, Ill.

It is an old custom in the legal and the medical professions to carry into their mutual relations a certain family feeling. Not that this feeling can be called particularly tender—just the average feeling that we meet with in families—but the professional presupposition of a certain blood connection.

A scientific analysis of either law or medicine does not bear out such presupposition; the law by having reference to medicine does not become different, and medicine, by being made a point in law, changes absolutely in no respect.

The existing connection is merely historical. It has its plain reason in the enormous importance of the medical expert in health questions, inner or outer. There was no congeniality which led the two professions together, but rather, what the old Romans, with reference to certain points in civil law, called "*communio incidens.*" Forensic medicine in our colleges is, therefore, no scientific category, but simply an expediency of didactics.

Hence, the necessity for the treatment of our topic, to investigate the scientific character of both branches of learning, their method of research and the position they occupy with reference to the leading features of scholarship of our time.

In proceeding with this proviso hither and thither, we must state, however, a very noteworthy difference between law and medicine. The latter is, of all the older sciences, the most progressive one; it adapted itself to the new discoveries in physics and chemistry, and is taking over from all other branches of learning whatever may promise fair to be an increase of enlightenment. Of the law, on the other hand, we must say that, if the historical part be taken away, there is nothing left which can in anyway claim to pass for a scientific element; it is nothing but a pile of arbitrary doctrine, theoretical sophistry, practically a perfect nuisance in the country—every country. Jurisprudence, as a study, is more than three thousand years of age, but it is, in its main features, what it was in the remotest centuries of civilization. The ideas of human society have changed considerably, practically, and are undergoing vastly more change in the mind of those who are the leading men in social development, theoretically. But the part which jurisprudence plays in it is the same as in darkest history. There is nothing in society, the knowledge of which has undergone so enormous mutation since antiquity, as *man*. In jurisprudence, the law—a man—precisely as thousands of years ago, is an individual with a name—the individual without change of identity, and the name in its constancy expressing this identity. Since jurisprudence was formed a glorious number of new sciences has arisen. Jurisprudence does not take note of them. All the intellectual world is in motion. There is, as it were, a quiver of intelligence, going through the universe. Jurisprudence is callous opposite the movement; its conservatism is putrid, its inner spirit of actuation stands stock-still; in its most brilliant reminiscences of history it exhibits a lethargy which dulls the burnish in which has consisted its splendor, and in its practical function it shows the consequences in loss of prestige and respectability.

The more serious representatives of the legal profession complain that the younger members—the new generation—are not careful any more to uphold the high standard of the profession so tenderly hugged by the veterans, and this is most true; these younger members lack the self-feeling required to make an effort; they admit that the law, that jurisprudence is on an inclining plane; but their professional honor is not roused by the menacing landslide towards disrespectful lines; they let go, and they go too.

An inquiry into the causes of this decay reveals its remote fountain in the nature of its origin and constitution. The law is the upshot of the rough-and-tumble of society, and jurisprudence, forming before a scientific standard was erected either high in or extending far to other branches of learning, in order to get a doctrinary outfit, bade over-eagerly welcome the occasion to fall back on its own dialectic resources, to accomplish its authoritative thought-construction by a sort of boomerang-argumentation, and up to this date there is in all philosophy of law not any law-thought, but it is a thought of law. It cannot be imagined in all method of erudition a greater burlesque than the doctrine, that the law, as soon as promulgated, must be considered to be known to everybody. In the entire world there is no judge, no attorney, that knows the laws of his own county, and if he knew them, his knowledge would be disputed by some other lawyer. But the theory of jurisprudence, at its methodological outset, is the fiction of that knowledge, and nobody may excuse himself with ignorance about the duly promulgated law. Nor is it only methodologically that jurisprudence used its own authority to become authoritative; it became ethical by stealth too. "*Qui suo jure utitur neminem lædit,*" says the ancient Roman jurisperitus, and all the other law-systems have the same axiom; whoever makes use of his right hurts nobody; in other words, no matter how wrong the law is to which you refer; by being law it becomes the right.

There has been in all political history nothing more demoralizing and indirectly more devastating than this ethical self-sufficiency of the law. All iniquity, all oppression, all disenfranchisement, took from there its legal connivance; and there is to-day no more brutal negation of justice and civilization than the superannuated axiom, whoever uses his right hurts nobody. The whole sophistry of modern hypocrisy is fomented by this argument, the flint-heart is fostered by it, which the jurisprudence deriving from it wants to be estimated by.

Jurisprudence is an altogether fictitious reality by time-worn doctrines. Never, in whatsoever state of society, in full accord with the idea of humanitarian aggregation, it is at present so altogether in contradiction to the essentialities of its requirements, that we may say, there is absolutely nothing so fulsome as it—the ridicule of civilization. Historically it was meant to come even out with justice. The ancient Roman law-philosophy never went beyond a confounding of *jus* and *justitia*, and conservative tongue flatters yet the law with the honoring interpretation. But there is no greater mistake than to believe *jus* and *justitia* synonyms. Still less is justice the outcome of the law, or, in the conditions of its existence, dependent on it. The law, so far from being the fountain of justice, is the surest sign of the absence, the default, of justice. People go to law when they will not listen to justice, either one or both. A lawsuit wants always to secure certain advantages against encroachment by antagonistic aspirations of a similar kind, no matter whether right or wrong. The law is individually settling, singling the one out in preference to another one; and all the vicious habits in society which result from egoism have the forensic contest as their favorite haunt and battle-ground.

"So many laws argue so many sins."—*Milton.*

The just man, on the other hand, needs no law. He is willing to view his right from the standpoint of his adversary, and to him there is no litigious point which gives occasion to a lawsuit. Justice supersedes the law. Or is not the degree of civilization necessarily determined according as a society needs law or can do without it? Have we ever seen the law promote virtue? Is it the tenderest, the poetically most exalted friendship which begins with a contract about the mutual standing? Is it in court—the euphemistically called courts of justice—where the matrimonial love is cultivated which henceforward keeps the married couple away from court? Does filial love start in the penitentiary? Must a man study the criminal code first in order to keep from manslaughter and murder?

There was never a greater mistake than to believe that a people is by the law educated into habits of justice. If there are habits of justice in activity, there is no occasion for law, and if we want to reform the law, the first step to do is to regard it in its true light, as a most pitiable makeshift of justice, which we must be anxious to exchange for the *spontaneity of doing positive good.*

All the law ever did, or will do, and all the law ever can do, is to prohibit the bad. It is made for the rogues, and by the law it is the good who are made bad, sooner at any rate than ever by it the bad have been made good. A law which would command the husband to love his wife would be a gratuitous item, to all intents and purposes only a countenancing of the grimaces of the youngster in law-practice. The law knows of no ideal goal.

If the law does anything on the moral field at all it is no more than to teach to assume the habit of never minding moral points in the relations of life.

It was by stealth only, and certainly undeservedly, that the law made itself at home in the honorable prestige of being the stand-by of civic morality. There are no theoretical, no scientific vouchers for the validity of the claim, and all the endeavor of the centuries, in untiring reiteration by enthusiastic admirers of the law, to prop jurisprudence as the prop of morality have fallen flat. Is it not, then, enough, after thousands of years of vain trial, to recognize practically the evanescence in flagrant bankruptcy of all moral assets, admitted by the law-scholars themselves, and to give up finally the law-fight as the only means of getting the peace of virtue?

"But there must be law!"

Yes, there must. Alas the day, it must! But let there be no more than can be helped, and let there be none, absolutely none, as a refuge of virtue and ennoblement! These will never thrive in such environment. Let it be, on the contrary, understood in public opinion, in legislation, and consequently also in the forensic *modus vivendi*, that the citizen who insists on a law-fight is *ipso facto* of an inferior cast, not aiming at the highest pitch of moral standing, but exerting himself to take advantage of clumsy doctrine *per fas et nefas*, by hook and by crook, applied in his favor. For the law, in its most inner nature, can never be anything else than such a taking advantage. It is in its very original idea the effort to secure to oneself's profit certain points which constitute exclusion of others, no matter how by such arrangement these others fare. As the world goes, when a party goes to law, it is not a question of justice, but one of race-chance: "What can I get out of it?"

If at least the law, in setting out to realize justice, did its very best to come near it! But all it does and ever has done is to do its very best to mar justice. If there had been hope yet for the trodden-down to be done justice to, once at the atrium of justice, or that which should be this, the halls of the protection of the right against the wrong, the word of Dante held:

> "Lasciate ogni speranza, voi ch'entrate."
> (Leave all hope behind, ye who enter here.)

The first dame of honor, it may be said, of the law, is Peace. In grim resentment of an offense of its fictitious majesty the court comes down with powerful empiricism on all obstreperous disregard of its harlequinade of forensic dignity; under the caption, contempt of court, another one of the letters of credit which jurisprudence had the wily shrewdness to write out for itself, the farce of legalized brutality has been made solemn by ceremony, and noble exasperation killed out among the antagonists of despotical arbitration. But what are these courts which are so jealous of the explosion of the truth to be held in contempt? They are the prostitution of justice; they institute in the forms of forensic etiquette the very same fight which they want to arbitrate. Jurisprudence is proud of the enormous reform which took place in the law when the ordeal was abolished; when a lawsuit was not decided any more by club and mace; when with the parties the brutal force was replaced by the power of reasoning about what was right and wrong; when the verdict of the judge did not depend on the question who was the more apt to knock the other's brain out, but on his own discrimination. But is not the sophistry-wrangle, which the attorneys do in the interest of their clients, the same old wrestle under a new name and in a modern form? Is not the circumstance that this wrestle wants to be paid virtually the continuance of the ordeal, only less romantic, coming on the contrary down to the dirty standard of justice being cried out to the highest bidder out of mere mercenary promptings?

The supremest virtue in a magistrate, the virtue without which no judge ought to be found, is Equity. Now, why not this same standard in the counsel? Can there be no equity in the judge, except the counsel have in iniquity first a fight over it?

With the old Romans, already, it was an ingrained maxim of the genius of the forum, that the judge had to hear both sides of the question; this was, so to speak, the elementary axiom of all jurisprudence: "*Audiatur et altera pars*" was the expression of it in their idiom; now, let us hear the other fellow; it was admitted that without such impartiality there could not be justice; and likewise in our days, as a principle, in all arbitration, it is conceded, that there can be neither truth nor justice without such impartiality. But is it not a fact that in the legal counsel we have an arrangement which is practically a slap in the face of this principle? Is it not the understanding, when attorneys are given to the parties to do for them the fighting about right, that they devote themselves, not to the detection of this right, as such, but exclusively to the interest which the party that pays them has with regard to it, and that they violate with all their cunningness an application of the maxim to the case in hand, seek to prevent it in the judge, even, and make efforts, with might and main, to get a verdict in direct contradiction to what the maxim would insinuate and suggest, warn and impress?

Truth, in our forensic custom, is elevated on the pedestal of civic virtue, or, so high that one has all the trouble in the world to find it; the witness, with a certain pomp of ceremony, about which the contempt-of-court farce is very particular, even, is exhorted to tell the truth, nothing but the truth, and all the truth. But the attorney is excused. This exception is his professional tickle, even; he is anxious to avail himself of the privilege to be exempt of the onerous duty, and if he does not tell downright lies, all the lies and nothing but lies, or is any way solicitous to abide by the truth, this is not due to any influence of the professional position, or to any restriction of his privilege to lie, but because his interest urges him on to be not quite so shameless as the forensic privilege would have him, or gives him liberty to be, but because his interest urges him on to do no more lying than is compatible with the aim he has in view. Lying is not always or in every respect the best thing to do, in court, even. So, the attorney does no more lying than is compatible with his hope to make his lying effective. He goes simply by the old proverb, that in a liar we do not estimate the truth, even. But he is sure to avail himself of his privilege as much as it is convenient and remunerative, and this condition of things continues and subsists, nay, flourishes, right into the face of the public claim of the courts to be the wardens of civic virtue, the stand-by of good order in society, the first and foremost foundation of civilization, and the means not only but the aim of progress towards public bliss. The great, celebrated theologian, Schleiermacher, said, if it had not been for the law, Christianism would never have secured civilization. The illusion!

To what end, besides, this lying? Is there no other way of getting at the truth in the judge, except by the lies of the attorneys?

In the discussion of special cases of encounters of physicians, as experts, and lawyers, as attorneys of the contending parties, usually the neglect of proper forms of dispute by the latter, is scored by the physicians as a personal solecism. This is a misinterpretation. The fault lies at the door of the system. The single lawyer is driven to the assumption of the disputing habits of the street arabs by the necessity he is placed under to make a living out of the odds of legislation. There is no possible reason, sensibly, for a separation of bench and bar; neither practically nor theoretically one can imagine, judicially or forensically, a sound motive for a line of demarkation between bar and bench. The attorney has the same vocation as the judge; it is the vocation to mark the boundary within which may move opposed will in society that appeals to the organs of the unity of the latter for decision. Thus why not give him the same position? Why make the attorney an outlaw, as it were, in court?

If the attorney were in the same dignity as the judge, the expert-physician would find no difficulty in dealing with the cross-examining counsel. Both would be imbued with the scientific object in hand, and their language would be mutually civil and dignified. There would be hither and thither a serious topic, nor any other ambition than to *detect the truth.*

There are adversaries to a proposition like ours who claim that by it the liberty of discussion would be restricted. There is no fact such an opinion could be fairly based on. Liberty of discussion stands or falls with the independence of the position of the party in question. Now, here the necessary proviso is in the hands of the people. It should be made as independent in the case of the attorney as in that of the judge, so that there would be nothing to exercise an unfavorable influence, nothing short of corruption, well, and that is something, no device, no institution can ward against. When the people itself is corrupt, then there is nothing in the people to stop such corruption. But if the like was to fear, we should have it in the judiciary now. The only difference would be, that with the independence of the legal counsel, not only from the State authorities but also from the private parties, the discussion in court, the pleading, would not be rant and rattle as it is so often to-day, but scientifically digestible and appreciative stuff, that which commands respect, sense and reason, TRUTH.

The first consequence of a salarying of the attorneys and their independence thereby from the parties whose case they handle would be an enormous diminution of lawsuits. Ninety-nine out of every hundred law cases can be easily compromised, if the parties were so disposed; the overwhelming majority of complications would never be made cases of, if it was not for the encouraging sicking by the lawyer who wants something to do. In this country more particularly, where the number of lawyers is exorbitant, the influence of an abolition of the institution of fee lawyers would draw from under the feet of the ruinous practice of the cultivation of the litigiousness the very bottom, and compel the incumbents to look out for some more useful occupation. There would be millions saved from the sums expended by the States for the administration of justice. But far exceeding the waste for forensic arrangements would be the enormous saving by economy of time and energy from the useless expenditure in cavil and irritating dispute, in pitiable alienation of the mind from gratifying and remunerative professional pursuit.

There is no reform more urgent than that of jurisprudence, the law. If the first French revolution failed so ignominiously, being delivered up, bound hand and foot, to their *bourreaux*, Robespierre, on the one hand, Napoleon, on the other, this was for no other reason than its incapacity to cope with the law-question. Among its intellectual authors, men like Voltaire and Jean

Jacques Rousseau, many were bold enough to brave the purgatory, but none had enough temerity to face in cold blood the eventuality to run society without the law. Hence the relapse of civilization for another long term into the night of the prejudicial ignorance of justice by law. It is high time to strip this prejudice. If there was in the people not a spring of virtue without the law, the law could never make it run, and if by law a line is drawn to the bad, it is practically only a check of the good which might have been done beyond the line of demarkation. Let there be law, if then it must! But let it be understood, that the higher standard is the avoidance of the law by acting the justice which supersedes it, and that virtually the law is only for the inferior minority, inferior in morality and numbers, whose nature is too mean to go by any other standard than that of judiciary and police force.

We know of a Western State (we think it is Nebraska), where the State society ruled out all ethical codification, as unethical, because insinuating a character which of the members should not be expected. Why cannot on a large scale be done what succeeds in a medical society? Why cannot an appeal like that of Admiral Nelson, "I expect of every man that he will do his duty," hold as well with reference to a nation, to humanity, as with that to an army? Why not with reference to the highest aim of man, if with that to a battle, an interest of most ambiguous civilization?

There is no surer way of anticipating the gentleman, than by presupposing in public opinion the actuality of his existence.

---

ACCORDING to the lay press, the Internal Revenue Bureau has decided that a physician or a druggist who prescribes for his patients whisky, brandy, wine or any other alcoholic liquor that is not compounded into a medicine by the admixture of any drug or medicinal ingredient therewith, is required to pay a special tax as a retail liquor dealer, even though the alcoholic liquor thus furnished be prescribed as a medicine only, and so used. This ruling is both unnecessary and unjust, and one, we trust, that can not be enforced.—*Courier of Medicine.*

